# Dermatology within the UK podiatric literature: a content analysis (1989-2010)

**DOI:** 10.1186/1757-1146-6-21

**Published:** 2013-05-24

**Authors:** Ivan R Bristow, Alan M Borthwick

**Affiliations:** 1 Faculty of Health Sciences, University of Southampton, Southampton, SO17 1BJ, UK

**Keywords:** Dermatology, Podiatry, History, Content analysis

## Abstract

**Background:**

Although dermatology, as a medical subject, has been a facet of the training and education of podiatrists for many years, it is, arguably, only in recent years that the speciality of podiatric dermatology has emerged within the profession. Some indication of this gradual development may be identified through a content analysis of the podiatric literature in the UK, spanning a 21 year timeframe.

**Method:**

6 key professional journals were selected for content analysis in order to provide a picture of the emergence and development of podiatric dermatology over a period extending from 1989 to 2010. Both syntactical and thematic unitization were deployed in the analysis, revealing both manifest and latent content. Categories were devised using a prior coding, a codebook produced to define relevant concepts and category characteristics, and the coding scheme subject to an assessment of reliability.

**Results:**

1611 units appeared in the 6 journals across a 21 year timeframe. 88% (n = 1417) occurred in one journal (Podiatry Now and its predecessors). Modal categories within all journals included course adverts (n = 673), commercial adverts (n = 562) and articles by podiatrists (n = 133). There was an overall rise from 40 per annum in 1989, to over 100 in 2010. A wider range of dermatological topics were addressed, ranging from fungal nail infections to melanoma.

**Conclusions:**

It is evident from this analysis that there has been an increasing focus on dermatology as a topic within the main podiatric journals in the UK over the last 21 years, primarily reflecting a rise in commercial advertising and an increase in academic dermatology related publications. Whilst earlier publications tended to focus on warts and fungal infections, more recent publications address a broader spectrum of topics. Changes in prescribing rights may be relevant to these findings, as may the enhanced professional and regulatory body requirements on continuing professional development.

## Background

Throughout the history of chiropody/podiatry in the United Kingdom, links to dermatology are evident within the professional literature. As a subject, it is evident as part of the UK chiropody curriculum since the inception of the first school in 1919 – initially as a subject delivered by visiting dermatologists and physicians and latterly, following the formation of the Society of Chiropodists, when formalization of the syllabus occurred in the 1950’s [1-3].

At a postgraduate level, for a period of 30 years (from 1945) the journal entitled “The Chiropodist” a professional podiatric journal of the Society of Chiropodists & Podiatrists had published over 50 papers on the subject of dermatology [4]. Virtually all of these papers were written by eminent dermatologists of the time such as MacKenna [5], Bettley [6], Ryan [7], Grant-Peterkin [8], Champion [9] and Williams [10]. Moreover, all of the papers were reprints from medical journals. As an editorial explained at the time, this was a sad reflection of the lack of research publications from within the chiropody profession [11] during this period. Records from this period demonstrate that dermatologists were also frequently invited to speak at Annual Conventions of the Society [12-16].

In 1975, the Society of Chiropodists had recognised the number of specialist interest groups emerging with a common interest in progressing technical skills (particularly podiatric surgery). As a consequence, a Postgraduate Board was assembled that year, consisting of representation from the Faculty of Anaesthesia, Podiatry Association and the Royal College of Physicians, to review and approve courses and as a means of ensuring quality standards, patient safety and insurance cover [17,18]. At the end of the decade and into the early 1980’s, chiropodists were increasingly being recognised as developing specialities in the care of the diabetic foot [19], ischaemic limbs [20] and “biomechanics” [21] facilitated by the increase in postgraduate courses available [22]. Towards the end of the 1980’s, development of these specialities were well established, in part, possibly driven by the new degree status awarded to the profession around this period [23]. A survey conducted in 1987 examined the expectations of chiropody students, and high numbers had aspirations upon graduation to work within these specialities [24].

Today, dermatology is taught at an undergraduate level both formally in the classroom and in the clinical setting. As with the medical model, demands for encompassing a wide range of areas in the professional curriculum have tended to side line the subject [25] despite the fact that skin problems on the foot are a common entity [26,27] and much of a clinicians work is focussed on treating skin related pathologies. Expanding roles within podiatry, as evidenced by the recent acquisition of rights to independent prescribing of medicines in both the UK and Australia, alongside a growth in other effective management therapies for disorders of the skin new to podiatry, further emphasise the need for good diagnostic and therapeutic skills. This paper aims to profile the development of dermatological knowledge within the UK podiatry profession, subsequent to the introduction of degree programmes in podiatry for a period of 21 years (1989-2010).

## Methodology

In order to build up an objective picture of the development of dermatology within podiatry data was obtained from published documentary evidence. Professional journals could be determined as covering a number of functions as outlined by Lundberg [28], providing a rich source of documentary evidence:

1. Provide peer-reviewed scientific and clinical evidence.

2. Hold an educational role.

3. Maintain practitioners’ awareness of contemporary issues within the profession.

4. Inform practitioners about non-clinical issues including politics, ethics and history.

5. Forecast future trends.

6. Provide basic background in science and research.

7. Act as a bridge between the textbook and the clinic.

The research questions were as follows:

1. What is the extent and coverage of dermatology within the selected journals (1989-2010)?

2. How has dermatological coverage changed during the study period?

Journals included for analysis consisted of the mainstream professional literature, available to UK registered chiropodists/podiatrists during the periods given below. Although relevant articles are published in other journals, the publications included here are recognized by professionals and are clearly identified within the field. Therefore, it should be expected to reflect, singularly and more accurately, the development of dermatological practice in chiropody/podiatry:

1. Podiatry Now (and its former titles - The Chiropodist and The Journal of British Podiatric Medicine): 1989 – 2010

2. The Foot (commenced in 1991): 1991 – 2010

3. Search News (ceased publication in 1997): 1990 – 1997

4. British Journal of Podiatry (Commenced 1998 and ceased publication in 2007): 1998 – 2007

5. Journal of British Podiatric Medicine & Surgery (ceased publication in 1997): 1989 – 1997

6. Journal of Foot and Ankle Research (E-journal commenced 2008): 2008-2010

The journals given above were subject to a well-established and widely applied method of documentary analysis known as content analysis [29]. Both a syntactical and thematic form of unitization was undertaken, centred on identifying keywords which could easily be replicated. Syntactical unitization is the identification of specific keywords which require little interpretation or judgement as to their meaning – for example words such as “dermatology” or “skin”. Such terms are relatively simple and are less open to interpretation and therefore more reliable [29]. Thematic unitization allowed for analysis of text identifying the present of a concept or theme which may not be overtly evident [30], as being part of the terms of study such as “bullous Impetigo” or “sun awareness and the risk of skin cancer” for example. By combining both forms of unitization it permitted the content to be explored with greater depth for validity and understanding within a reference frame of dermatology and ensured that both manifest and latent content were assessed [31].

Categories were devised in a manner as described by Weber [32] using a technique of a priori coding [33] where categories were determined prior to the analysis. In order to gain context to the data, categories were constructed by the author to be meaningful, reflecting the different aspects of the journal function, for example:

•Editorial: Reflecting the political views of the professional body.

•Correspondence: Expressing the issues of the readership.

•Advertisements: Illustrating current trends in industry.

•Articles by Podiatrists: Current areas of publications and research.

•Conference Abstracts: Representing special interest groups.

•News: Current issues of political, professional or personal interest.

To reduce ambiguities, a codebook was produced clearly defining the concept under analysis and individual category characteristics. The coding scheme was then tested by analysing a sample of text. This was undertaken to verify that the categories held a clear definition ensuring exclusivity and that all data was exhaustive.

Following the testing stage, each journal in the specified range was read and any reference or commentary related to dermatology was noted and placed into one of the categories. The timeframe across which this study ran was determined as twenty-one years, reflecting the time the author has been registered.

Inclusion criteria for the analysis included any themes relating to skin disorders affecting the feet. Themes were excluded if they related to routine palliative procedures for nails, corns and callus as implied in the definition given by Farndon et al. [34] as this represents a function of “core” podiatry/chiropody skills. To prevent overlap into areas of wound care and diabetes care any themes related to common diabetic skin manifestations (vascular and neurological processes) were excluded from the analysis. Coding of the data were undertaken exclusively by the authors.

### Intercoder reliability

To ensure basic stability of the coding scheme, an assessment of reliability was undertaken employing the use of a second (independent) coder trained in content analysis – a process termed reproducibility [35]. An entire journal was purposively selected and coded by the second coder to test reproducibility. This journal represented around 8.5% sample of the total items coded.

The data from the two coders was then calculated using Krippendorff’s alpha (α) statistic. Although many different tests are available, this test is widely used and considered robust, taking into account chance agreement and, in addition, the magnitude of disagreements, adjusting also for the type of variable type (nominal, ordinal etc.,)[35-37] which is not addressed by other reliability measures [38].

The Krippendorff’s alpha statistic ranges from .00 (no agreement) to 1.00 (perfect agreement) demonstrating a high level agreement from the data. Krippendorff’s alpha (α) statistic was calculated using the SPSS Statistics Package version 17 (SPSS Inc, USA) and the customized SPSS macro for Krippendorff’s alpha [37]. Reproducibility for this data were reported as 0.9727 - very high, indicating a high level of agreement between coders.

### Findings

**Figure 1:**
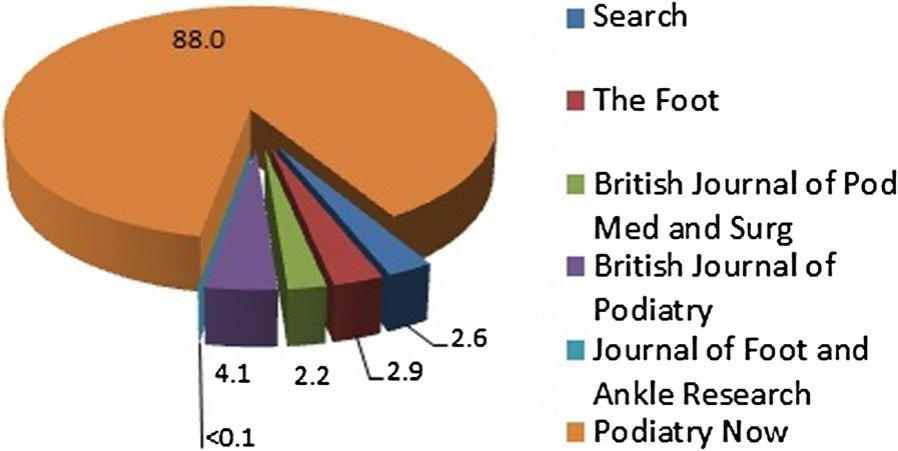
Total journal share (%).

In the period from 1989-2010, a total of 1611 units appeared in the 6 journals analysed. The division of the units across the publications is given in Figure 1. Of these 88.0% (n = 1417) occurred in one journal - Podiatry Now (and its former titles) with just 12.0% (n = 194) spread across the remaining five journals.

**Figure 2:**
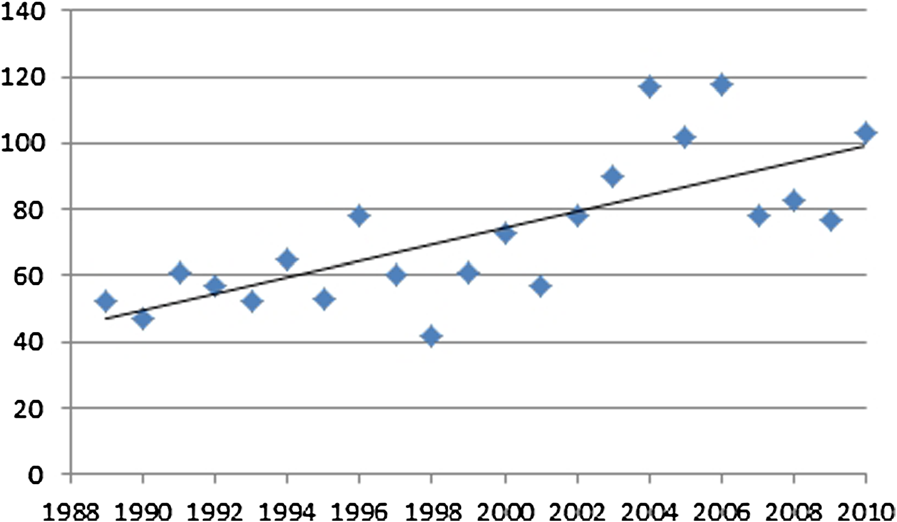
Total references to dermatology (1989-2010).

The modal categories within all journals were course advertisements/reports (n = 673) followed by commercial advertisements (n = 562) and articles by podiatrists (n = 133). Over the study period a gradual increase in the number of references to dermatology was shown to rise from an average of around 40 per annum in 1989 to over 100 in 2010 (Figure 2).

### Search journal

The journal “Search” ran for 7 years from 1990 until being integrated into Podiatry Now [39] and was circulated primarily to managers within the NHS consequently little dermatological content was evident. In the latter half of its publication years, a number of short news items were published including reporting trials from medical research on warts [40], psoriasis [41], melanoma [42] and new products [43]. Only three dermatological papers appeared in the journal written by podiatrists; on the topics of fungal nail treatments [44], urea based emollients [45] and dry skin [46] and were generally small NHS departmental studies. Latterly, mentions of the dermatology were made when discussing the development of continuing professional development programmes in 1996 [47].

### The British journal of podiatric medicine and surgery

The British Journal of Podiatric Medicine and Surgery (BJPMS) was the official journal of the Podiatry Association (PA), published up until 1997 when it became part of the British Journal of Podiatry [39]. The PA was predominantly a group representing the interests of podiatric surgeons within the UK. Despite its surgical leanings, a small number of references to dermatological papers published in medical journals are made on topics such as onychomycosis and melanoma [48]. The topic of antifungals and steroid creams also occurs when discussions are made on prescription rights in 1996 [49]. As expected papers on nail surgery are evident, albeit in small numbers (n = 3) in the 7 year period.

### The foot journal

The Foot (fully titled “The Foot – the International Journal of Clinical Foot Science”) differed from all the other podiatry journal analysed in this study as it is the only one which the editorial board is shared with another professional group; orthopaedic surgeons. The journal was launched in 1991 and was originally distributed with Society of Chiropodists members’ subscription free of charge until around 2001. It was hoped to showcase graduate research and become an academic journal with Index Medicus status [39,50] and establish an impact factor - something at the time which had eluded all UK podiatric journals [51]. Analysis of its content revealed a total of 32 dermatological related papers. Of these 14 were published by medics and focussed primarily on skin tumours and common foot infections [52-65] whilst podiatry lead author papers focused on mycoses [66-71], wart infections [72-74], electrosurgery [75,76] and epidemiology [77]. Interestingly, other single papers by Podiatrists focused on less commonly encountered dermatological conditions [78-82]. Three papers were investigations into the pathology of corns and callus [83-85]. Nail surgery was a topic of eight papers [86-93]. Minimal course advertisements and commercial advertising were evident in this academic publication.

### British journal of podiatry

The British Journal of Podiatry began in 1998, comprising of the two former journals – The Journal of British Podiatric Medicine and the British Journal of Podiatric Medicine and Surgery intending to carry editorials, refereed articles and professional information. In its 10 year history, 20 dermatological papers were identified written by Podiatrists with an additional 4 on nail surgery [94-97]. Areas of dermatological focus included warts [98-101], fungal infections [102,103], case studies on tumours and unusual infections [104-110]. Four studies looked at more academic areas and laboratory work relating to dermatology [111-114]. Two papers looked at treatments for skin conditions [115,116].

### Journal of foot and ankle research

Less than 0.1% of all units occurred in this journal, explained by its three year lifespan during the study period. Only five publications were evident in the online journal. Four these were on the subject of melanoma, submitted by the author [117-120] whilst one paper studied the epidemiology of callus patterns in the elderly [121].

### Podiatry Now

**Figure 3:**
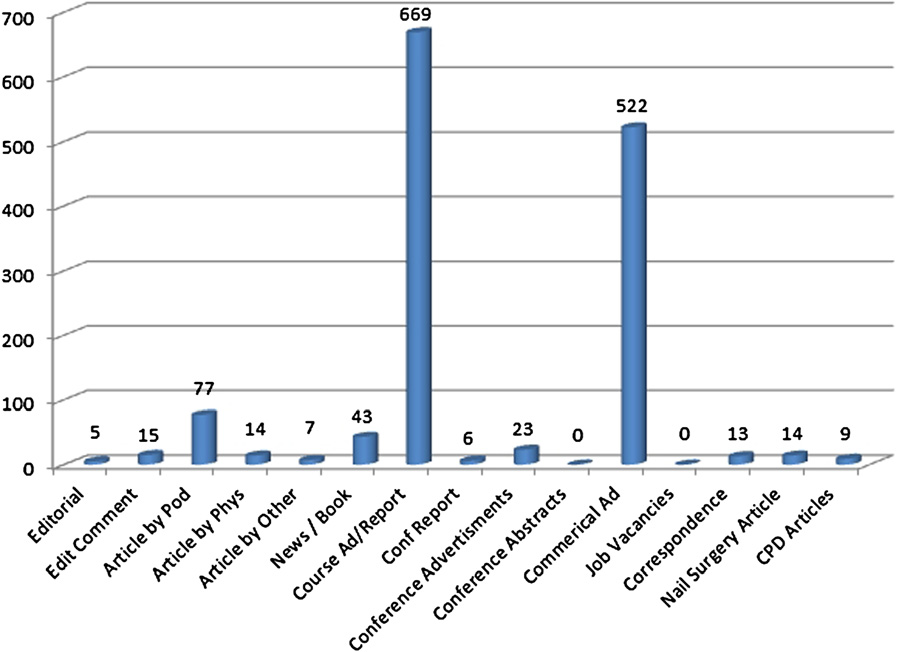
Frequency of units by category in podiatry now (1989-2010).

This journal represented the vast majority of units (1417, 88%) within this analysis. In part this may be explained by its longevity compared to the other journals - none of which covered the entire period of study but also a reflection of the functions and audience types of each of the journals analysed. Podiatry Now as a professional journal covered many of the categories identified in this study when compared to the other publications which tended to focus more on academic papers only or one particular aspect of practice – podiatric surgery for example. The modal categories, within this journal, were course advertisements (699) and commercial advertisements (522) over 21 years. A full breakdown is given in Figure 3.

Throughout the twenty-one years, 77 dermatology related articles, authored by podiatrists, have been published in the journal. The publication rate has remained at a constant level averaging around 3-4 a year. The focus of the articles has varied slightly over this period. In the earlier years the attention has been on traditional skin problems such as warts [122-125], fungal infections [126-128] as well as general articles on common dermatoses such as psoriasis [129,130]. At the end of the 1990’s although papers on the traditional topics were still being covered [131-133], skin tumours became an interest with case studies emerging on melanoma [134], squamous cell carcinoma [135,136] and Bowen’s disease [137]. At this time, inclusion of a section entitled “Clinical Picture Quiz” which invited authors to send in a clinical quiz for readers attracted a number of dermatological cases over the subsequent years [138-140] including eight from the author [141-148].

Physicians and other healthcare professionals published 14 and 7 dermatology related articles respectively in the journal. The majority of physician authored articles were reprints from other medical journals (n = 9) [149-157]. The reasons for this are unclear. It may reflect a lack of suitable podiatric literature during this period on the subject or an editorial decision to include broader knowledge which may be relevant to podiatrists.

Throughout the study time frame, editorial commentary and correspondence on dermatological themes were rare, particularly in the latter half of this period. In the early part of the period studied, discussion of various dermatological topics in editorial comment was mainly through the writing of Colin Dagnall. Dermatological topics were briefly discussed in his column in the journal (simply entitled “Dagnall”) which ran until the December 1992 issue but also latterly by Dagnall through the correspondence pages. A few years on, references were made in the editorial about dermatological conditions such as fungal foot infections, warts and their treatments and were mentioned in the journal as part of the medianote section as well as occasional new product announcements. A small increase of the study period was also noted for news items and book reviews with an average of one unit per year in 1989, rising modestly to around 3 per year by 2010.

**Figure 4:**
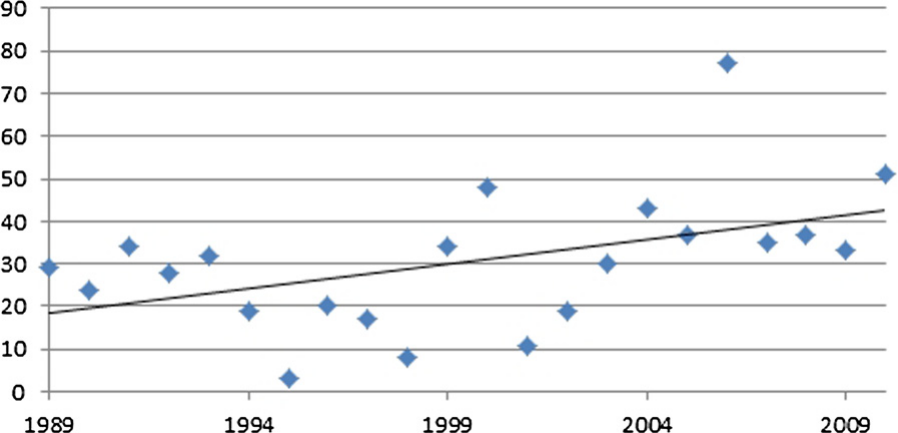
Course advertisements and reports in podiatry now (1989-2010).

Course advertisements on dermatological topics and reports from the data demonstrate a significant increase with time (see Figure 4), on average, doubling during the study period from 20 to over 40, whilst nail surgery courses had virtually ceased.

The increase in the number of course advertisements could be explained by a number of possible factors. First, as the Society has grown in its membership, the number of local branches has increased along with the amount of activity. To accommodate this, the journal may have increased the advertisement of locally run courses. More likely however, was the political climate and the Governments move to improve health care in the late 1990’s. The paper entitled ‘A First Class Service’ [158] and subsequent communications [159] outlined the need for continuing professional development (CPD) within health care professions to maintain and improve quality as part of the clinical governance initiative [160]. The response from the Society of Chiropodists and Podiatrists was the launch of a programme of CPD modules in 2001 for its members [161] including a dermatology module, written by the author [162], which became a part of the core modules offered in 2003 [163]. Latterly, the introduction of mandatory CPD was placed on chiropodists and podiatrists with the introduction of the Health Professions Council (HPC) biennial audit cycles which began in 2006 [164]. Prior to the changes in the CPD process in 2003, only eight individual study days on dermatology had been advertised as update courses to members in the preceding 14 years but following the introduction of the topic as a core subject the numbers of advertisements for such courses increased dramatically to 15 courses in the following 7 years. This trend was mirrored by an increase in the number of course reports which were published in the journal.

The outlier observed in Figure 4 (77 course advertisements in 2006) represented a year in which a large number of dermatological courses were being run whilst simultaneously being heavily advertised. The reason for this is not clear. However, increased advertising in this year may be a reflection of the introduction of mandatory CPD by the Health Professions Council, influencing many local SCP branches to increase their educational provision for their members during this particular year. Similar but smaller increases were noted for the years 1999 (34) and 2000 (48). Both of these represent a disproportionate amount of advertising by an independent company for a course in electrosurgery. Other courses offering dermatological skills (cryosurgery) were identified. Both electrosurgery and cryosurgery courses were more frequently advertised in the first half of the study period suggesting that the need (or demand) for training in these modalities had decreased.

In 2004, inserts appeared within the journal Podiatry Now. These were published as update articles on various topics to enhance the availability of CPD to members [165]. Up until the end of 2010, nine articles had been published with a dermatological theme including fungal infections [166,167], warts [168], psoriasis and eczema [169], emollients [170], cryosurgery [171], electrosurgery [172], melanoma [173] and non-mechanical causes of hyperkeratosis [174].

**Figure 5:**
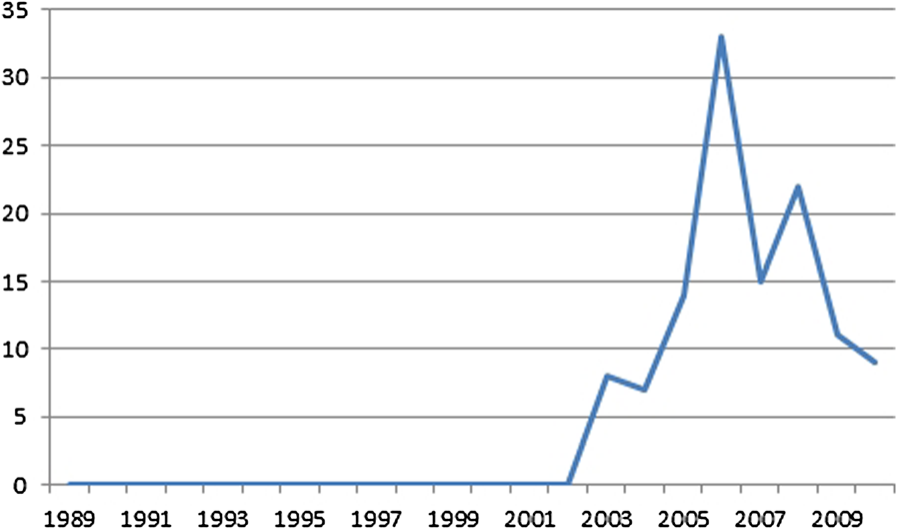
Frequency of distance learning advertisements in podiatry now (1989-2010).

**Figure 6:**
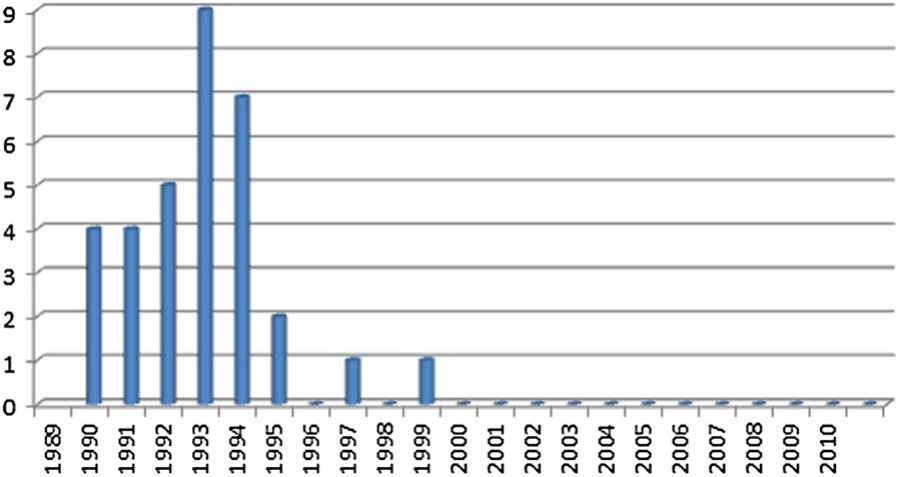
Nail surgery course advertisements in podiatry now (1989-2010).

As part of the CPD process, distance learning modules in dermatology were noted to be advertised monthly within the journal, offered by a private company from 2003 continuously until the end of the study period (Figure 5). It is assumed that such courses were advertised as an alternative means for podiatrists to receive CPD, in particular for those in private practice who maybe geographically isolated or unable to travel to conferences and similar events. The steep rise in advertisements around 2005 maybe explained, once again, by the introduction of mandatory CPD for podiatrists at this time. The demise of the nail surgery course (Figure 6) could be explained as training in nail surgery techniques only became part of the undergraduate curriculum around the early 1980’s, following the introduction of local anaesthesia [175]. This meant many chiropodists/podiatrists who qualified before this period would be required to undertake an approved course to acquire the appropriate skills. By 2000, one could assume that all those requiring training would have sought it during this period and hence there was no need to run further courses.

**Figure 7:**
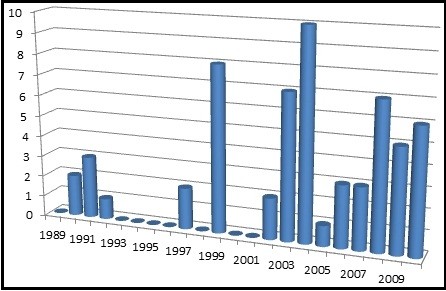
Podiatrist delivered lectures (1989-2010).

Over the study period, physicians were providing the majority of dermatological lectures within podiatry (n = 118), with nearly twice as many advertisements as Podiatrists (n = 60) although an analysis of frequency counts suggested an increasing trend for more Podiatrists delivering lectures (Figure 7).

Many lectures by doctors were delivered at a local branch level by consultant dermatologists across the UK. The most frequent topics delivered were general dermatology of the foot and skin tumours/melanoma, when stated in the advertisement. The first conference dedicated wholly to podiatric dermatology was advertised in 2003 [176] and took place at the University College, Northampton. The conference ran for four years on annual basis and accounted for the conference advertising evident in the data from 2003 to 2006.

“Lectures by others” was a category used to denote advertised lectures on dermatological subjects not given by a podiatrist or physician (or occasionally, when the designation of the speaker was not stated). The majority of these were presentations on aspects of microbiology (particularly fungal infections) delivered by mycologists. In addition, some related to prescription only medicines listed as given by a “company representative” (for products such as Loceryl®, Dovonex® and Sporanox®) occurring across the whole of the study period.

Despite the raised profile of the dermatology within podiatry, it has rarely been advertised as a higher education module until recently – a total of five advertisements, with four being in the period 2007-2010 with modules offered by Universities across the UK (Universities of Stirling, Salford, and Sunderland with Glasgow Caledonian University and Queen Margaret University, Edinburgh).

### Commercial advertising in podiatry Now

**Figure 8:**
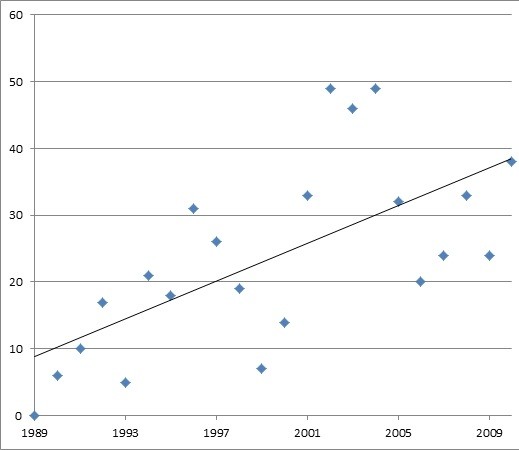
Frequency of commercial advertising in podiatry now (1989-2010).

Throughout the 21 year period, analysis of the amount of dermatology related advertising demonstrated an increase in number products advertised to podiatrists. This can be seen as a few products advertised in 1989, rising to around an average of 40 annual advertisements by 2010 (see Figure 8).

Initial advertisements focused on cryosurgical equipment but this is quickly overtaken in two years by the promotion of emollient products focused on the foot. These continue to be the major part of dermatological advertising in the latter part of the study period. Other frequent products include topical salicylic acid (Occlusal®) preparations, topical antifungal and antiseptic agents (Mykored®, Crystacide® and Monphytol®). The majority of the advertising occurred within Podiatry Now which could be explained as its role as a professional journal reaching all members of the professional body, advertisers would be well aware of its circulation over the other journals mentioned. Particularly heavy periods of advertising are noted in the period 2002 – 2004 which is a reflection of a few emollient and antifungal products being profoundly advertised within this period. The reasons for this are unclear.

### Prescription only medicines

In 1994, the first prescription-only medicine (POM) was advertised to UK podiatrists within Podiatry Now. The drug Terbinafine (Lamisil®) is an oral antifungal agent used in the treatment of tinea pedis and onychomycosis. Although UK podiatrists are not able to prescribe independently (just yet), access or supply this medication, the advertisement was carried. The motives of the pharmaceutical company are not clear but presumably this was based on the assumption that podiatrists encounter a lot of fungal foot disease and may consider referring on cases to general practitioners. Subsequently, this would actively promote the fact that a medication was available and ultimately raise awareness and prescriptions of the drug through the general practitioner. Following on in 1996, Itraconazole (Sporanox®) and Amorolfine nail lacquer (Loceryl®) other prescription only medicines were included in the same journal and ran several marketing campaigns. To accompany this, a number of evenings were advertised in the journal, hosted and presented by pharmaceutical representatives promoting antifungal agents and treatments for psoriasis (Dovonex®) to podiatrists. Despite the move in recent years towards increased access to medicines and prescribing, with the introduction of patient group directions and supplementary prescribing for podiatrists, no other advertisements for prescription only medicines have appeared in the journal.

### Limitations of the study

Content analysis is defined as a research technique for making replicable and valid inferences from texts (or other meaningful matter) to the contexts of their use [35]. Neuendorf [177] defines it as the systematic, objective, quantitative analysis of message characteristics. As a technique it can be useful to discover and describe the focus of social attention [32]. The technique allows the researcher to sift through large quantities of data systematically allowing the discovery of inferences and the examination of patterns and trends in documents [33]. This content analysis aimed to give an overview of dermatology and its development within podiatry for the period 1989-2010. In undertaking this, measures were taken to ensure that the data were representative and reliability tests were been undertaken to assess the coding procedure. A full analysis of the mainstream podiatry journals was conducted, rather than a sample, for this period in an attempt to capture the most complete picture

It must be acknowledged that this data can only represent what has been published within these journals and may not fully present the true picture. For example, many courses which have been run during this period may not have been advertised using this medium and so will not have been included. In addition, frequency counting as the sole form of analysis can be misinterpreted, therefore attempts have been made to further analyse the data in order to describe and explain any findings. Content analysis is inherently reductive when placing the data into categories. When defining boundaries and definitions for the key terms it is accepted that there will be material which could be considered borderline. The definition which has been adopted as it emphasizes core podiatry skills – the routine palliative procedures for nails, corns and callus acting as a delineation from dermatology. To prevent overlap into areas of wound care and diabetes any themes related to common diabetic skin manifestations (vascular and neurological processes) were excluded from the analysis. Subsequently, papers have been excluded although it could be argued contribute in a broader sense to dermatology and podiatry including the work of Springett [178], Potter [179,180] and Hashmi [113,181] along with much of the literature pertaining to the diabetic foot.

## Conclusions

This content analysis set out to profile the coverage of dermatology within the mainstream podiatric literature from 1989-2010. From this analysis it is evident that there has been an increasing focus on dermatology as a topic within the main podiatric journals published in the UK. This has been predominantly as a rise in advertising of commercial products and increase in dermatology related publications. Nearly 90% of all references to dermatology appeared in one podiatry journal – Podiatry Now and its predecessors. Analysis of the papers published in the journals has shown work was predominantly authored by podiatrists and physicians, with a proportion of the latter featuring as reprints from other journals. Earlier publications from podiatrists tended to focus on traditional skin disorders of warts and fungal infections but more recently case studies and papers have emerged on other skin conditions. Educational and CPD opportunities in dermatology have increased in line with the changes to HPC re-registration and professional body CPD requirements.

## Competing interests

Both authors declare that they have no competing interests.

## Authors’ contributions

IB was responsible for the original design of the study, along with initial data analysis and the drafting of the manuscript. AB acted as second analyst of data and contributed to subsequent drafts of the paper. All authors read and approved the final manuscript.

## References

[B1] Ford HP The development of chiropody The Chiropodist 1951 6 325 334

[B2] Read PJ Education in chiropody The Chiropodist 1964 19 49 53

[B3] Rosenstein H The future of chiropody in the national health service The Chiropodist 1959 14 49 58

[B4] Bristow IR The origins and evolution of podiatric dermatology, PhD thesis 2011 Southampton, United Kingdom: University of Southampton, Faculty of Health Sciences

[B5] MacKenna RMB Diseases of the skin in the elderly The Chiropodist 1966 22 266 276

[B6] Bettley FR Effects of soap on the skin The Chiropodist 1959 14 304 311

[B7] Ryan TJ Vasculitis The Chiropodist 1978 33 11 16

[B8] Grant-Peterkin GA Common skin infections of the feet The Chiropodist 1947 2 98 100

[B9] Champion RH Diseases of the skin - treatment of psoriasis The Chiropodist 1968 23 113 119

[B10] Williams DI Treatment of fungal infections by griseofulvin The Chiropodist 1961 16 149 154

[B11] Anon Be sure of your facts…(editorial) The Chiropodist 1959 14 41 42

[B12] Anon Liverpool convention 1969 The Chiropodist 1969 24 22 25

[B13] Anon Silver jubilee convention The Chiropodist 1970 25 98 102

[B14] Anon The 1966 cambridge convention in retrospect The Chiropodist 1966 21 185 186

[B15] Anon The norwich convention The Chiropodist 1960 15 351 354

[B16] Anon Forthcoming events and conventions The Chiropodist 1956 11 97 98

[B17] Anon Postgradute education (editorial) The Chiropodist 1976 31 81 82

[B18] Anon Postgraduate education The Chiropodist 1975 30 245 246

[B19] Hockaday TDR Improving and extending chiropody services The Chiropodist 1979 34 337 338

[B20] Anon Diabetes The Chiropodist 1980 35 421 422

[B21] Anon Biomechanics The Chiropodist 1983 38 331 332

[B22] Anon The future (editorial) The Chiropodist 1984 39 2 3

[B23] Spencer C Degree courses for chiropodists The Chiropodist 1988 43 131 133

[B24] Marsh A An investigation into the attitudes and expectations of final year chiropody students with particular reference to the national health chiropody service The Chiropodist 1989 44 108 113

[B25] Bristow IR Springett K Podiatry, dermatology & the UKPDA British Assoc Dermatol Newsletter 2006 9 22 23

[B26] Burzykowski G Molenberghs D Abeck E Haneke E Hay RJ Katsambas D Roseeuw D van der Kerkof P van Aelst R Marynissen G High prevalence of foot diseases in Europe: results of the achilles project Mycoses 2003 46 496 505 10.1046/j.0933-7407.2003.00933.x 14641624

[B27] Thomson J A dermatology service in a school of chiropody The Chiropodist 1978 33 100 101

[B28] Lundberg GD The role and function of professional journals in the transfer of information Int J Technol Assess Health Care 1988 4 51 58 10.1017/S0266462300003251 10287115

[B29] Krippendorf K Content analysis. An introduction to its methodology 1980 London: Sage

[B30] Sproule W Walter M Content analysis Social research methods: an australian perspective 2006 Melbourne: Oxford University Press 113 134

[B31] Kondracki NL Wellman NS Amundson DR Content analysis: review of methods and their applications in nutrition education J Nutr Educ Behav 2002 34 224 230 10.1016/S1499-4046(06)60097-3 12217266

[B32] Weber RP Basic content analysis 1990 London: Sage

[B33] Stemler S An overview of content analysis Pract assess res eval 2001 17 7

[B34] Farndon L Vernon W Potter J Parry A The professional role of the podiatrist in the new millennium:an analysis of current practice. Paper 1 Br J Pod 2002 5 68 72

[B35] Krippendorff K Content analysis:an introduction to its methodology 2004 2 London: Sage

[B36] Krippendorff K Estimating the reliability, systematic error, and random error of interval data Educ Psychol Meas 1970 30 61 70 10.1177/001316447003000105

[B37] Hayes A Krippendorff K Answering the call for a standard reliability measure for coding data Commun Methods Meas 2007 1 77 89 10.1080/19312450709336664

[B38] Lombard M Snyder-Duch J Bracken C Content analysis in mass communication: assessment and reporting on intercoder reliability Human Commun Res 2002 28 587 604 10.1111/j.1468-2958.2002.tb00826.x

[B39] Anon The camden accord - the publications (editorial) J British Podiatric Med 1997 52 169 170

[B40] Anon Wart treatment by doctors Search 1995 68 9

[B41] Anon Diabetes versus psoriasis Search 1995 62 6

[B42] Anon What’s new on the melanoma front? Search 1995 60 9

[B43] Anon Psoriasis - new treatment products Search 1993 57 6

[B44] George MA A comparison of three treatments for fungal nail infection Search 1991 16 7 8

[B45] Kerr T Clinical trial of dermal therapy Search 1994 47 4 6

[B46] Fisher A Hay S The motivation of the elderly with a problem of dry feet Search 1995 62 3

[B47] Joyce P Post graduate podiatry education the oxford personnel training plan (PTP) Search 1996 73 7

[B48] Anon Podium British J Podiatric Med Surg 1995 7 72

[B49] Galloway TR Extension of access provision for a limited list of prescription only medicines (editorial) British J Podiatric Med Surg 1996 8 19 20

[B50] Anon International journals (editorial) Br J Pod 1999 2 102

[B51] Borthwick AM ‘Publish and be damned’:disseminating contemporary podiatric research Br J Pod 2004 7 91 92

[B52] de Berker D Dawber RPR Diagnostic twists in neoplasms of the nail apparatus Foot 1992 2 73 77 10.1016/0958-2592(92)90021-G

[B53] Smith AG Inflammatory dermatoses of the feet Foot 1992 2 195 198 10.1016/0958-2592(92)90048-T

[B54] Perks AGB Miller G Watson JS Acral lentignous malignant melanoma in a diabetic foot:a plea for better education Foot 1993 3 83 85 10.1016/0958-2592(93)90067-D

[B55] Smith AG Skin tumours of the foot Foot 1994 4 175 179 10.1016/0958-2592(94)90048-5

[B56] Heim M Primary squamous carcinoma of the foot: a missed diagnosis Foot 1996 6 74 76 10.1016/S0958-2592(96)90040-1

[B57] Walzack JP Klugman DJ Squamous cell carcinoma of the hallux Foot 1997 7 33 10.1016/S0958-2592(97)90009-2

[B58] Montgomery F Fioriti A Piezogenic papules:treated by resection and hernial closure Foot 1998 8 171 172 10.1016/S0958-2592(98)90055-4

[B59] Heim M Wershavski M Siev-Ner I Azaria M A late complication of erysipelas Foot 1999 9 203 205 10.1054/foot.1999.0565

[B60] Smith AG Skin infections of the foot Foot 1999 9 56 59 10.1054/foot.1999.0515

[B61] Thoolen M Ryan T Bristow I A study of the skin of the sole of the foot using high-frequency ultrasonography and histology Foot 2000 10 14 17 10.1054/foot.1999.0568

[B62] Ramlogan D Batta K Byrne J Pemphigus vulgaris presenting as chronic osteomyelitis of the foot Foot 2001 11 74 75 10.1054/foot.2001.0668

[B63] Tytiun Y Iordache S Grintal A Velkes S Salai M Bacterial skin contamination and bacterial recolonization, after surgical preparation, in foot operations and prevalence of postoperative early wound infection: a prospective study Foot 2005 15 74 76 10.1016/j.foot.2005.02.003

[B64] Barkham MC Smith AG Harris S Eccrine syringofibroadenoma Foot 2006 16 198 200 10.1016/j.foot.2006.07.003

[B65] O’Neill AC Purcell EM Regan PJ Interdigital pilonidal sinus of the foot Foot 2009 19 227 228 10.1016/j.foot.2009.04.002 20307483

[B66] Mann GC Berry BL Inopportune choice of treatment for verruca pedis Foot 1996 6 80 81 10.1016/S0958-2592(96)90042-5

[B67] Nicolopoulos CS Tsioutis V Nicolopoulos NS Giannoudis PV Clinical application of helium neon (632 nm) plus infrared diode laser GaAIAs (830 nm) and CO2 laser in treatment of onychomycotic nails Foot 1999 9 181 184 10.1054/foot.1999.0527

[B68] Ricketti JC Terbinafine/miconazole nitrate 2% tincture compound for the treatment of onychomycosis Foot 2001 11 21 23 10.1054/foot.2000.0653

[B69] Davies KJ Study to determine the efficacy of Clotrimazole 1% cream for the treatment of onychomycosis in association with the mechanical reduction of the nail plate Foot 2006 16 19 22 10.1016/j.foot.2005.10.004

[B70] Zalacain A Ruiz L Ramis G Novel V Ogalla JM Calvo MA Vinuesa T Podiatry care and amorolfine: an effective treatment of foot distal onychomycosis Foot 2006 16 149 152 10.1016/j.foot.2006.03.007

[B71] Zatcoff R Smith MS Borkow G Treatment of tinea pedis with socks containing copper impregnated fibers Foot 2007 18 136 141 10.1016/j.foot.2008.03.005 20307427

[B72] Bristow I Walker N Pulsed Dye laser for the treatment of plantar warts - two case studies Foot 1997 7 229 230 10.1016/S0958-2592(97)90042-0

[B73] Powell J Papillomavirus research and plantar warts Foot 1998 8 26 32 10.1016/S0958-2592(98)90016-5

[B74] Ellis MJH Thomson CE Consumer health information on the WWW: an evaluation of information on verrucae Foot 2003 13 130 135 10.1016/S0958-2592(03)00010-5

[B75] Bevans JS Bosson G A comparison of electrosurgery and sharp debridement in the treatment of chronic neurovascular, neurofibrous and hard corns. A pragmatic randomised controlled trial Foot 2010 20 12 17 10.1016/j.foot.2010.03.003 20434674

[B76] Anderson JM Burrow JG A small-scale study to determine the clinical effectiveness of electrosurgery in the treatment of chronic helomata (corns) Foot 2001 11 189 198 10.1054/foot.2001.0686

[B77] Mølgaard C Lundbye-Christensen S Simonsen O High prevalence of foot problems in the danish population: a survey of causes and associations Foot 2010 20 7 11 10.1016/j.foot.2010.03.002 20382520

[B78] Vohra S Bates AW Baithun SI A rare adnexal tumour of the hallux:malignant chondroid syringoma Foot 1996 6 175 177 10.1016/S0958-2592(96)90017-6

[B79] Padhiar N Necrobiosis lipodica diabeticorum Foot 1997 7 47 51 10.1016/S0958-2592(97)90013-4

[B80] Barlow A Parry E Role of foot orthoses in the management of epidermolysis bullosa simplex Foot 1999 9 60 64 10.1054/foot.1999.0510

[B81] Anderson PJ Maloney DJL Berry RB Subcutaneous granuloma annulare Foot 2000 10 40 41 10.1054/foot.1999.0556

[B82] Smith RG Shoe dermatitis: a review of current concepts Foot 2008 18 40 47 10.1016/j.foot.2007.12.001

[B83] Potter J Potter MJ The effect of callus removal on peak plantar pressures Foot 2000 10 23 26 10.1054/foot.2000.0576

[B84] Potter J Potter MJ Regrowth patterns of plantar callus Foot 2000 10 144 148 10.1054/foot.2000.0599

[B85] Springett KP Whiting MF Marriott C Epidemiology of plantar forefoot corns and callus, and the influence of dominant side Foot 2003 13 5 9 10.1016/S0958-2592(02)00112-8

[B86] Dagnall JC A description of toenail matrix phenolisation 44 years before Boll’s 1945 paper Foot 1991 1 51 55 10.1016/0958-2592(91)90014-3

[B87] Paul AS Ohiorenoya B Meadows TH Subungual exostosis presenting as an ingrowing toenail Foot 1991 1 125 126 10.1016/0958-2592(91)90021-3

[B88] Goslin RW A comparison of dilution and non-dilution of phenol with alcohol following nail avulsions Foot 1992 2 225 228 10.1016/0958-2592(92)90052-Q

[B89] Middleton A Webb F Toenail surgery for diabetic patients Foot 1993 3 109 113 10.1016/0958-2592(93)90076-F 1511580

[B90] Heim M Cohen I Blankstein A Hakerem D Missre A Dudkiewicz I To ablate or not to ablate, that is the question Foot 2001 11 97 98 10.1054/foot.2001.0673

[B91] Rounding C Hulm S Surgical treatments for ingrowing toenails Foot 2001 11 166 182 10.1054/foot.2001.0692 10796808

[B92] Kaleel SS Iqbal S Arbuthnot J Lamont G Surgical options in the management of ingrown toenails in paediatric age group Foot 2007 17 214 217 10.1016/j.foot.2007.06.007

[B93] Córdoba-Fernández A Ruiz-Garrido G Canca-Cabrera Á Algorithm for the management of antibiotic prophylaxis in onychocryptosis surgery Foot 2010 20 140 145 10.1016/j.foot.2010.09.007 20961749

[B94] Davies KJ Case report: pseudomonas aeruginosa infection following nail avulsion Br J Pod 2000 3 7 9

[B95] Blake A A postoperative comparison of partial nail avulsion using phenol and cryotherapy Br J Pod 2005 8 128 132

[B96] Cumming S Stewart S Harborne D Smith J Broom H Abbott A Barton A A randomised controlled trial of phenol and sodium hydroxide in nail surgery Br J Pod 2005 8 123 127

[B97] Day JC A retrospective comparative study between HIV positive individuals and HIV negative individuals who have undergone partial nail avulsion surgery for onychocryptosis Br J Pod 2006 9 60 62

[B98] Chapman C Visaya G Treatment of multiple verrucae by triggering cell-mediated immunity - a clinical trial Br J Pod 1998 1 89 90

[B99] Lelliott PE Robinson C A retrospective study to evaluate verrucae regrowth following electrosurgery Br J Pod 1999 2 84 88

[B100] Forster M The use of electro-desiccation in the treatment of corns and verrucae Br J Pod 2000 3 64 66

[B101] Mortimer A Percivall A A review of english senior school policies for the prevention and spread of verrua plantaris Br J Pod 2001 4 95 100

[B102] Bristow I Onychomycosis - a review of its presentation and treatment Br J Pod 2004 7 64 67

[B103] Brown LA MacLarnon N Do patients with untreated tinea pedis have concomitant fungal contamination within their footwear and hosiery Br J Pod 2007 10 134 138

[B104] Swinscoe M Podiatric parasitology Br J Pod 1998 1 106 108

[B105] Weir HK Carline T Nail changes associated with the human immunodeficiency virus and acquired immunodeficiency syndrome-a case report Br J Pod 2000 3 9 12

[B106] Wall B Leishmaniasis - an overview of the disease and two case studies Br J Pod 2001 4 3 7

[B107] Bours PW Gilheany MF Pyogenic granuloma. An atypical appearance in the foot Br J Pod 2007 10 57 59

[B108] Hutchby M Metcalfe S Digital adenocarcinoma Br J Pod 2007 10 45 46

[B109] Ward R Boon G Case report. Lesser toe fibrolipoma in a patient with hereditary angioedema Br J Pod 2007 10 35 38

[B110] Waddington AM Hogg L The surgical management of a subungual acral malignant melanoma:a case presentation and review of the literature Br J Pod 2007 10 166 170

[B111] McCourt FJ Normal plantar stratum corneum and callus. An analysis of fatty acids Br J Pod 1998 1 98 101

[B112] Ingle M The structure of keratin Br J Pod 1999 2 13 16

[B113] Hashmi F Non-enzymatic glycation and the development of plantar callus Br J Pod 2000 3 91 94

[B114] Plummer J Springett K Phillips G Skin protection for use with caustics in podiatric practice: efficiency of a polymer paint Br J Pod 2004 7 68 70

[B115] Wilkinson A Kilmartin T A study into the long term effectiveness of electro-surgery for the treatment of corns Br J Pod 1998 1 138 141

[B116] Rankin S Swinscoe M Alternative treatments and folk remedies in the treatment of warts Br J Pod 2002 5 12 14

[B117] Bristow I Acland K Acral lentiginous melanoma of the foot: a review of 27 cases J Foot Ankle Res 2008 1 11 10.1186/1757-1146-1-11 18822168 PMC2553782

[B118] Bristow I de Berker D Development of a practical guide for the early recognition of malignant melanoma of the foot and nail unit J Foot Ankle Res 2010 3 22 10.1186/1757-1146-3-22 20920168 PMC2954980

[B119] Bristow IR Bowling J Dermoscopy as a technique for the early identification of foot melanoma: a review J Foot Ankle Res 2009 2 14 10.1186/1757-1146-2-14 19435498 PMC2694773

[B120] Bristow IR de Berker DA Acland KM Turner RJ Bowling J Clinical guidelines for the recognition of melanoma of the foot and nail unit J Foot Ankle Res 2010 3 25 10.1186/1757-1146-3-25 21040565 PMC2987777

[B121] Spink M Menz H Lord S Distribution and correlates of plantar hyperkeratotic lesions in older people J Foot Ankle Res 2009 2 8 10.1186/1757-1146-2-8 19331676 PMC2669075

[B122] Custance B Clinical case study Chiropodist 1990 45 148 150

[B123] Morris HJ The treatment of multple and mosiac warts Chiropodist 1989 44 2,4

[B124] Russell M Podiatric cryosurgery Chiropodist 1990 45 195 197

[B125] Ashford R Steele K Granuflex E hydrocolloid dressing and Salactol in the treatment of verruca pedis:a randomised comparative study J British Podiatric Med 1993 48 107 113

[B126] George MA A comparison of three treatments for fungal nail infection Chiropodist 1991 46 134 135

[B127] Foxall JD Richards N A review of podiatric management of onychomycosis (tinea unguium) J British Podiatric Med 1993 48 53 56

[B128] Mooney J A review of current treatments for toenail mycoses J British Podiatric Med 1993 48 5 6

[B129] Kingsford AC Psoriasis Chiropodist 1989 44 35 39

[B130] Beeson P The clinical significance for chiropodists of recent advances made in the pathology and treatment of Psoriasis Chiropodist 1990 45 43 46

[B131] Santos D Pseudomonas aeruginosa infection of the nail:a case report British J Podiatric Med 1997 52 37 38

[B132] Stepney E Fungal nail infection - a new perspective Podiatry Now 1998 1 221 223

[B133] Swinscoe MJ A case of onychomadesis associated with the use of co-trimoxazole in a female patient - a clinical report J British Podiatric Med 1997 52 30

[B134] Thomson T Malignant melanoma and squamous cell carcinoma on the foot. Four case reports J British Podiatric Med 1996 51 149 150

[B135] Haverstock B Squamous cell carcinoma of the foot - a literature review and case report British J Podiatric Med 1997 52 76 79

[B136] Sandilands S Bligh MG Subungual squamous cell carcinoma in the toe. A case report J British Podiatric Med 1996 51 5 6

[B137] Stewart GA Case study Podiatry Now 1998 1 415

[B138] Kaura C Francis BE Clinical picture quiz 54 Podiatry Now 1999 2 343

[B139] Kilmartin T Clinical picture quiz 43 Podiatry Now 1998 1 295

[B140] Conway JJ Clinical picture quiz 36 Podiatry Now 1998 1 7 12

[B141] Bristow I Clinical picture quiz Podiatry Now 2004 7 26 30

[B142] Bristow I Clinical picture quiz Podiatry Now 2007 10 46

[B143] Bristow IR Clinical picture quiz Podiatry Now 2003 6 13 34

[B144] Bristow I Banfield CC Clinical picture quiz 47 Podiatry Now 1999 2 87

[B145] Bristow IR Clinical picture quiz 58 Podiatry Now 2000 3 191 211

[B146] Bristow I Clinical picture quiz 61 Podiatry Now 2000 3 468 472

[B147] Bristow IR Clinical picture quiz 64 Podiatry Now 2001 4 569

[B148] Bristow I Case study challenge (dermatology) Podiatry Now 2002 5 117

[B149] Danci T Ersanli Z Skin and connective tissue disorders in diabetes mellitus J British Podiatric Med 1996 51 151 154

[B150] Denning DW Evans EGV Kibbler CC Richardson MD Roberts MM Rogers TR Warnock DW Warren RE Fungal nail disease:a guide to good practice (report of a working group of the british society for medical mycology) J British Podiatric Med 1996 51 62 66 10.1136/bmj.311.7015.1277 PMC2551187 7496239

[B151] Williams HC Pottier A Strachan D The descriptive epidemiology of warts in British school children J British Podiatric Med 1994 49 171 176

[B152] de Berker D Management of nail unit wounds British J Podiatric Med 1993 48 175 177

[B153] Butcher W Papworth S Parvin S Darke S Eighty-five consecutive cases of cellulitis:clinical features, management and implications for hospital care Podiatry Now 2003 6 26 29

[B154] Tan BB Lear JT English JSC Metastasis from carcinoma of the breast masquerading as chilblains J British Podiatric Med 1997 52 143

[B155] Mattone-Volpe F Cutaneous larva migrans infection in the pediatric foot Podiatry Now 1998 1 289 293 10.7547/87507315-88-5-228 9610046

[B156] Cox NH Colver G Paterson WD Management and morbidity of cellulitis of the leg Podiatry Now 1999 2 292 295 10.1177/014107689809101206 PMC1296982 10730111

[B157] Baron S Goulden V Stables G Atypical melanoma Podiatry Now 2004 7 22 23

[B158] Department of Health A first class service - quality in the new NHS 1998 London: HMSO

[B159] Department of Health Continuing professional development. Quality in the new NHS 1999 London: HMSO

[B160] Department of Health Clinical governance - quality in the New NHS 1999 London: HMSO

[B161] Potter MJ Making progress with CPD and beyond (editorial) Br J Pod 2002 5 3

[B162] Bristow IR Core update course in dermatology 2003 College of Podiatrists, Society of Chiropodists & Podiatrists: Faculty of Podiatric Medicine

[B163] Potter M Faculty of podiatric medicine Br J Pod 2003 6 3

[B164] Anon HPC introduces CPD requirements for podiatrists from July 2006 Podiatry Now 2006 9 6

[B165] Potter MJ Continuing professional development and podiatry Now Podiatry Now 2004 7 22

[B166] Bristow IR Onychomycosis:guide to management Podiatry Now 2004 7 S1 S8

[B167] Bristow I Tinea pedis:diagnosis and management Podiatry Now 2004 7 S1 S8

[B168] Potter M Bristow I The treatment and management of verrucae using caustics Podiatry Now 2006 9 S1 S8

[B169] Bristow IR Ersser S Inflammatory skin conditions: psoriasis and eczema Podiatry Now 2004 7 S1 S8

[B170] Penzer R Emollients:selection and application Podiatry Now 2005 8 S1 S8

[B171] Colver G Cryosurgery in podiatric practice Podiatry Now 2005 8 S1 S8

[B172] Percivall A Principles of electrosurgery Podiatry Now 2006 9 s1 s8

[B173] Ardern-Jones M Malignant melanoma Podiatry Now 2008 11 s1 s8

[B174] Bristow IR Hyperkeratosis of the foot: part 1 Podiatry Now 2008 11 S1 S8

[B175] Anon Continuing professional development - a new initiative (editorial) J British Podiatric Med 1996 51 129 130

[B176] Bristow I Podiatric dermatology group to be launched at conference Podium 2003 1 10

[B177] Neuendorf KA The content analysis guidebook 2002 London: Sage

[B178] Springett K Parsons S Young M Cheek E The effect and safety of three corn care products Br J Pod 2002 5 82 86

[B179] Potter J The use of salicylic acid in the treatment of dorsal corn and callus Br J Pod 2000 3 51 55

[B180] Potter J Hyperkeratosis of the foot:part 2 Podiatry Now 2008 11 S1 S8

[B181] Hashmi F Malone-Lee J Hounsell E Plantar skin in type 2 diabetes:an investigation of the biochemical and biomechanical nature of plantar epidermis (poster) Br J Pod 2004 8 25

